# Renal protection in sepsis: Is hypertonic sodium (lactate) the solution?

**DOI:** 10.1186/s13613-019-0505-z

**Published:** 2019-02-08

**Authors:** Patrick M. Honore, Leonel Barreto Gutierrez, Herbert D. Spapen

**Affiliations:** 1 ICU Department, Centre Hospitalier Universitaire Brugmann/Brugmann University Hospital, 4, Place Arthur Van Gehuchten, 1020 Brussels, Belgium; 2 Ageing and Pathology Research Group, Vrije Universiteit Brussel, Brussels, Belgium

**Keywords:** Hypertonic sodium lactate, Resuscitation, Fluids, Acute kidney injury, Sepsis

Hypertonic sodium solutions are useful for minimizing secondary brain injury from cerebral edema but also attractive for “small-volume” resuscitation in severe hypovolemia and shock because they rapidly mobilize endogenous water from the intracellular reservoir, thereby reducing endothelial cell volume and restoring microcirculation.

In an exhaustive study in endotoxic pigs, Duburcq et al. [1] showed that early fluid resuscitation with hypertonic sodium lactate (HSL) attenuated disseminated intravascular coagulation-associated renal microvascular thrombosis and improved renal function. HSL performed significantly better than 0.9% NaCl [normal saline (NS)] and hypertonic sodium bicarbonate (HSB).

Two striking findings emanate from this study. First, resuscitation fluids substantially interfere with pathophysiological events that occur during sepsis. Fluids indeed have a direct and intimate contact with damaged and activated endothelium and may be involved in the early intricate endothelial-mediated inflammatory and coagulation processes that determine the course of sepsis (e.g., the intrinsic beneficial effect of 20% albumin infusion on skin endothelial function in septic shock patients [2]). The mechanism of action, however, remains hypothetical, clearly differs between fluids, and probably is multifactorial. Second, hypertonic solutions are not created equal. This is illustrated by the observation that HSB, despite equal amounts of sodium and similar alkalizing, osmotic, and anti-inflammatory effects as HSL, had no better effect on acute kidney injury (AKI) than NS.

The study of Duburcq et al. suggests a poor protective renal effect of NS in early sepsis. In recent years, NS has been increasingly stigmatized as an undesirable and even detrimental resuscitation fluid. However, this kidney-unfriendly profile of NS is not reflected in clinical scenarios. Yunos et al. [3] linked NS administration to the development of hyperchloremic metabolic acidosis and AKI. However, lactated/balanced crystalloids and 20% albumin were substituted for 4% albumin, gelatin, and NS according to an unblinded before–after protocol. Which of these changes contributed to the improved renal outcome and whether unidentified confounders may have influenced the incidence of AKI is unknown. Infusion with 0.9% NS versus Plasma-Lyte^®^ in healthy volunteers produced hyperchloremia and significant reductions in mean renal artery flow velocity and renal cortical tissue perfusion in the NS group. However, relevant AKI was not evidenced as shown by similar concentrations of an AKI biomarker in both infusion groups [4]. A meta-analysis assessing high- versus low-chloride infusion in 6253 perioperative and critically ill patients found a significant but weak association between high-chloride-containing fluids and a higher incidence of AKI. Excluding the 2 most-weighted studies from this analysis, however, rendered the AKI endpoint non-statistically significant [5]. A subsequent cluster crossover randomized clinical trial did not demonstrate differences in AKI in intensive care (IC) patients who received either NS or Plasma-Lyte^®^ [6]. The recent SALT cluster-randomized, multiple-crossover trial comparing the administration of similar volumes of NS and balanced crystalloids in 974 IC patients showed no difference in a composite “major kidney-related adverse events” endpoint including death, dialysis, and persistent renal dysfunction [7]. Importantly, none of the above studies could demonstrate an increased mortality rate in NS-treated patients.

Crystalloid fluids with lower chloride content and a strong ion difference, or the so-called balanced solutions, may contribute to a better clinical outcome. Zampieri et al. [8] reported lower hospital mortality and a reduced incidence of AKI from days 3–7 after IC admission in a large cohort of critically ill patients receiving Ringer’s lactate. This beneficial effect was more pronounced as the total administered volume increased. Surviving patients received proportionally more Ringer’s lactate and less NS than non-survivors. Of note is that only few septic patients were included. Most studies compared balanced solutions with NS. No study has evaluated the effects of hypertonic versus balanced fluid resuscitation on morbidity and mortality.

Scant evidence indicates that administration of hypertonic saline is associated with fewer perioperative complications, a shorter IC stay, and better survival [9]. Lactated hypertonic solutions additionally improve cardiac performance [10]. A French multicenter trial studied the effects of infusing no sodium bicarbonate versus 4.2% HSB to maintain pH > 7.30 in IC patients. Septic shock was present in 55% of HSB recipients. Each fluid was administered as a 30-min bolus infusion with a maximum of 1000 mL within 24 h. The probability of survival at day 28 between the control and HSB group was not significant. HSB-treated patients, however, had lower need for renal replacement therapy (RRT) and were less dialysis-dependent at IC discharge. HSB also decreased 28-day mortality in patients with more severe AKI [11]. The recently published HYPERS2S trial in mechanically ventilated patients with septic shock found no significant difference in organ failure and mortality between patients treated with 3% hypertonic saline or NS. Compared with NS-treated subjects, the hypertonic saline group showed a trend (*p* = 0.13) to less need for RRT. Results also argued against hyperchloremia, which was significantly more frequent in the hypertonic saline group, as a factor increasing the risk of developing AKI [12].

In their endotoxic shock model, Duburcq et al. [13] previously reported a lower cumulative fluid balance in HSL-treated animals as compared to the NS and HSB group. This was associated with significantly enhanced oxygenation, hemodynamics, cardiac performance, and microcirculatory function. In septic patients, maintenance and replacement fluids had a more absolute impact on fluid balance and sodium/chloride levels than resuscitation fluids [14]. Thus, it remains unclear whether clinical, and in particular renal, outcome depends on the use of a specific hypertonic resuscitation solution or on curbing an inappropriate fluid creep.

Concern also exists about the use of HSL. In an ovine model of septic shock, administration of HSL was associated with more pronounced decreases in blood pressure and cardiac function, earlier onset of impaired tissue perfusion, and shorter survival time [15].

We agree with Duburcq et al. that hypertonic, and particularly lactated, solutions deserve to be evaluated as resuscitation fluids, preferentially in a large randomized clinical trial enrolling carefully selected and homogenous patient groups, choosing the appropriate control fluid (balanced crystalloid rather than NS?), and assuring a thorough assessment of relevant outcomes and safety issues.

## Abbreviations

HSL: hypertonic sodium lactate
NS: normal saline
HSB: hypertonic sodium bicarbonate
AKI: acute kidney injury
IC: intensive care
RRT: renal replacement therapy
